# Case report: three ways to mitigate the risk of embolization during left atrial appendage closure in a patient with a massive and proximal left atrial appendage thrombus

**DOI:** 10.1093/ehjcr/ytae286

**Published:** 2024-06-11

**Authors:** Sandra Zendjebil, Jérôme Horvilleur, Victor Boilève, Vincent Millien, Philippe Garot

**Affiliations:** Institut Cardiovasculaire Paris Sud (ICPS), Hôpital Jacques Cartier, Ramsay-Santé, 6 avenue du Noyer Lambert, Massy 91300, France; Institut Cardiovasculaire Paris Sud (ICPS), Hôpital Jacques Cartier, Ramsay-Santé, 6 avenue du Noyer Lambert, Massy 91300, France; Institut Cardiovasculaire Paris Sud (ICPS), Hôpital Jacques Cartier, Ramsay-Santé, 6 avenue du Noyer Lambert, Massy 91300, France; Service de cardiologie, Centre hospitalier Saint Quentin, Saint Quentin, France; Institut Cardiovasculaire Paris Sud (ICPS), Hôpital Jacques Cartier, Ramsay-Santé, 6 avenue du Noyer Lambert, Massy 91300, France

**Keywords:** Case report, Left atrial appendage closure, Thrombus, Stroke, Device, Left atrial appendage

## Abstract

**Background:**

Left atrial appendage (LAA) thrombus is a contraindication for LAA closure (LAAC). However, in selected cases, oral anticoagulants are strictly contraindicated because of a history of life-threatening bleeding, and LAAC remains the only possible therapy to avoid systemic and especially cerebral embolization.

**Case summary:**

We report a case of LAAC despite a massive proximal thrombus in a patient who had an absolute contraindication to anticoagulant therapy, with thorough pre-planning using CT scan, device modelling and thrombus trapping techniques to reduce the risk of systemic embolic events and perform LAAC safely.

**Discussion:**

Although LAAC remains at high risk in this setting, the use of cautious techniques and tools, from pre-procedure planning to systemic embolization prevention systems associated to a precise transoesopheageal echocardiography guiding throughout the procedure, allows it to be performed as safely as possible when no other option is available.

**Learning points**

- To show that careful technique of left atrial appendage closure (LAAC) device implantation can be used in the presence of a proximal LAA thrombus with good transoesopheageal echocardiography guidance.
- To underline the interest of planning the procedure ahead, especially with CT scan and AI-enabled modelling, to facilitate a prompt implantation from the first attempt.

## Introduction

Left atrial appendage (LAA) thrombus is a contraindication to LAA closure (LAAC). However, in selected cases, oral anticoagulants are strictly contraindicated because of a history of life-threatening bleeding. We report a case of LAAC despite massive proximal thrombus, with precautions and techniques used to reduce the risk of systemic embolic event.

## Case presentation

A 74-year-old man was admitted for acute bilateral limb ischaemia in a context of permanent atrial fibrillation (AF). He was successfully treated with surgical thrombectomy and anticoagulation. However, after a fall, he suffered a head injury with a right subdural haematoma. Consequently, anticoagulants were transiently interrupted until a repeat cranial CT scan showed bleeding cessation. Nevertheless, the patient presented with right hemiplegia and a new CT scan revealed a new left intracerebral haematoma. Finally, anticoagulation was strictly contraindicated.

Ten years ago, the patient had suffered a myocardial infarction treated with a stent on the LAD and permanent AF complicated by ischaemic stroke. The CHA2DS2-Vasc score was 7, and the HAS-BLED score 5.

Despite several risk factors for peripheral artery disease, the fact that the lower limb ischaemia was bilateral and simultaneous favours an embolic mechanism in a setting of permanent AF. Thrombus usually migrates from the LAA, but in this patient, it could also have originated from the left ventricle after a previous myocardial infarction. Scenarios of a thrombus migrating from an ulcerated aortic plaque or resulting from coagulopathy are less likely. As the patient was on anticoagulation before limb ischaemia, compliance may have been suboptimal, or the dose underestimated.

Transthoracic echocardiography (TTE) showed preserved left ventricle ejection fraction, with limited anterior hypokinesia, bilateral atrial dilatation, and no valve disease, therefore reinforcing the hypothesis of thrombus migration from the LAA.

Laboratory tests revealed a haemoglobin of 10.7 g/dL (normal if >13 g/dL), a platelet count of 216 G/L (normal between 150 and 500 G/L), an estimated glomerular filtration ratio of 87 mL/min (normal if >120 mL/min), a fibrinogen of 2.8 g/L (normal if <4 g/L), and no inflammatory syndrome.

In this setting, to reduce the risk of recurrent ischaemic embolic event, a LAAC was scheduled, and the patient was referred to our centre.

**Figure 1:**
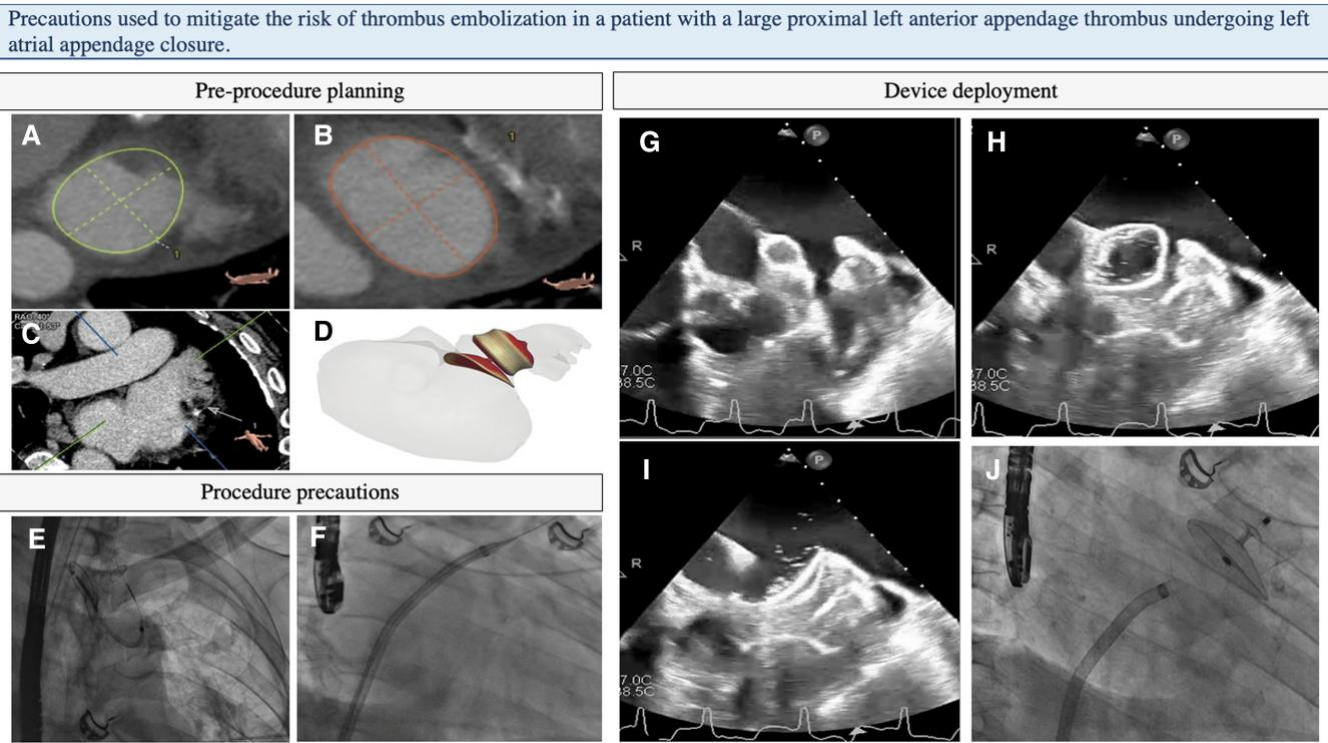
(i) Pre-procedure planning: cardiac CT-guided optimal sizing and transseptal planning to avoid supplementary manipulations and device mis-sizing. (*A*–*C*) 3Mensio™ analysis with diameters of landing zone and ostium of a cauliflower-shaped appendage: 27.4 × 32.2 mm (mean 29.8 mm) and 30.1 × 40.8 mm (mean 35.5 mm), respectively. (*D*) FEops HeartGuide™ simulation of a distal AMULET 34 mm device showing a good compression and ridge coverage. (ii) Procedure precautions: (*E*) sentinel device through right radial access. Total dose of heparin administered before transseptal punction. (*F*) Stiff wire and delivery sheath in the upper left pulmonary vein, no injection and no pig tail in the appendage to prevent thrombus embolization. (iii) Device deployment. (*G*–*I*) ‘No touch technique’: ball-shaped device entering the appendage without touching the thrombus, and instantaneous opening of the lobe and the disk to ensure total sealing. (*J*) Neither recapture, nor tug test.

First of all, pre-operative planning was critical to ensure a safe procedure. Pre-procedural cardiac CT scan revealed a cauliflower-shaped appendage, with an ostium and landing zone average diameters of 35.5 mm and 29.8 mm, respectively, and a depth of 26.2 mm. Distal appendage thrombus was suspected on the contrast-enhanced acquisition. We simulated different sizes and positions of AMULET (Abbott Vascular, IL, USA) device with the FEops HEARTguide™ (FEops, Gent, Belgium) AI-enabled simulation software to select the most appropriate size and position for this particular anatomy. A distal position of a 34 mm AMULET device was deemed to be the adequate combination with a 15% lobe compression, suitable ostium coverage, and sufficient device disc-lobe separation (*Figure 1*). Moreover, FEops HEARTguide™ also enabled us to predict the most adequate site for a transseptal punction to catheterize axially the appendage.

Under general anaesthesia, transoesopheageal echocardiography (TEE) confirmed LAA dimensions, but displayed a massive proximal appendage thrombus (*Figure 1*). Although it remains a contraindication to LAAC, the heart team decided to proceed with the procedure as the risk of further embolism was very high, and contraindication to anticoagulation therapy was strict, with no chance of safely rescheduling the procedure after decreased thrombus burden.

Several tools were therefore used to cautiously seal the appendage and the thrombus within. Femoral venous puncture was performed with prior closure, and the full dose of heparin (11 000 UI) was immediately infused (150 UI/kg) with a close Activated Clotting Time (ACT) monitoring (aiming at 350 s). A Sentinel™ device (Claret Medical, Santa Rosa, USA) was inserted in the brachioencephalic trunk and left carotid artery through a 6 F right radial access prior to transseptal puncture to decrease the risk of periprocedural stroke caused by thrombus migration. Transseptal puncture was cautiously performed under TEE and fluoroscopy guidance. Once the stiff wire had been introduced into the left superior pulmonary vein, the delivery sheath was inserted. Left atrial pressure was measured at 12 mmHg, and the 34 mm device was then loaded without contrast injection in the LAA. The device was unsheathed under permanent TEE and fluoroscopy guidance in the left atrium. The two operators thoroughly coordinated themselves to orient the ball-shaped device very anteriorly, to avoid posterior thrombus and to be able to cautiously advance the device into the LAA.

Once sufficiently deep, the lobe was deployed, and the disc immediately unsheathed to trap the massive thrombus between the lobe and the disc (*Figure 1*). Final lobe diameter was 29 mm, with a 15% compression, similar to that estimated by the simulation. As TEE showed no evidence of peri-device leak and complete ostium coverage, the device was released without prior tug test. The cerebral protection device revealed no thrombus, and the patient presented no neurologic defect when awakened from anaesthesia. He showed no neurological symptom or signs of systemic embolization on Day 3 and up to 3 months. Transthoracic echocardiography displayed a good seal of the appendage, with neither peri-device leak nor device-related thrombus, confirmed by the 3-month control CT scan (*Figure 1*). No anticoagulation or antiplatelet therapy was initiated due to the initial medical history.

## Discussion

Although the presence of a proximal thrombus in the LAA is a contraindication for LAAC, this case shows that the procedure may be performed when no alternative therapy is possible. Indeed, anticoagulation therapy either with heparin or direct anticoagulants would have been too risky given the patient’s recent history of iterative cerebral bleeding. A half dose of anticoagulants could have been discussed, but with unknown efficacy and a definite bleeding risk. Surgery remained contraindicated, leaving no therapeutic alternative other than LAA. However, when performed, this technique carries a high risk of thrombo-embolic event. Several precautions should be taken to ensure a safer procedure. The feasibility and safety of LAAC have already been described in cases of distal LAA thrombi (trapping technique, ‘no touch’ technique…) using a minimalist implantation technique with thorough imaging guidance.^1,2^

Firstly, using AI-enabled patient specific simulation as provided by FEops HEARTguide™ helps predict the appropriate size and position of the device, therefore limiting the need for device mis-sizing and the use of additional device that is associated with an increased rate of thrombus migration. It also predicts the appropriate transseptal puncture location, therefore defining the optimal route to the LAA and accelerating the procedure.

Secondly, tools and techniques to avoid both thrombus formation and embolization can be implemented. Full-dose heparin after femoral vein puncture, no appendage opacification and permanent TTE guidance to advance the ball-shaped prosthesis into the appendage are critical. In this case, the thrombus was located very proximally, with a free area allowing the ball-shaped device to be advanced in a controlled manner.

Finally, the no touch technique, meaning avoidance of any material or contrast injection inside LAA, reduces the risk of thrombus dislodgment. Additionally, efforts to implant the device from the first attempt should be maximal. Tug test were not performed due to the proximal location of the thrombus, lodged between the disc and the lobe. According to a recent systematic review, the use of the AMULET device in these patients would be safer because it has to be implanted less distally, and the disc usually can seal the LAA, reducing the risk of thrombus embolization.^3^

Scarce data are available on the usefulness of cerebral protection devices in such cases. Although they were inconsistently used in previously published cases^3^ and no thrombus was found in our case, thrombus parts and even cardiac tissue were captured with these devices in a small study.^4^ Systemic embolization is more accessible to interventional therapy, although embolization of the digestive arteries remains a dreaded complication. Hence, the use of a Watchman device in the aorta to prevent systemic embolization has previously been described.^5^

In conclusion, this case report indicates that a bailout LAAC with a proximal, massive LAA thrombus may be feasible when no therapeutic alternative is available, subject to dedicated additional precautions that include meticulous CT planning with adequate device simulation, appropriate procedural precautions to prevent embolic events with adequate anticoagulation and a cerebral protection device, and subsequent ‘trapping technique’ for device deployment.

## Lead author biography


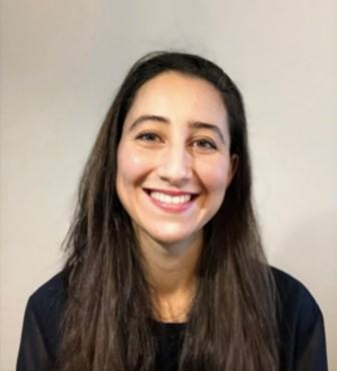


Dr Sandra Zendjebil is an interventional cardiologist at Hôpital Bichat, Paris, France, and was a fellow in interventional cardiology at the Institut Cardiovasculaire Paris Sud (ICPS). She has a particular interest in structural heart procedures, in particular in valvular diseases and left atrial appendage closure.

## Supplementary Material

ytae286_Supplementary_Data

## Data Availability

The data underlying this article cannot be shared publicly to preserve the privacy of the patient involved in this case report. The data will be shared upon reasonable request to the corresponding author.

## Notes

**Consent:** Informed consent was obtained to use anonymized data in line with COPE guidance.

**Funding:** None declared.
